# Interactions of biotic and abiotic environmental factors in an ectomycorrhizal symbiosis, and the potential for selection mosaics

**DOI:** 10.1186/1741-7007-6-23

**Published:** 2008-05-28

**Authors:** Bridget J Piculell, Jason D Hoeksema, John N Thompson

**Affiliations:** 1 Department of Ecology and Evolutionary Biology, 1156 High Street, University of California, Santa Cruz, Santa Cruz, CA 95064, USA; 2 Department of Biology, University of Mississippi, University, MS 38677, USA

## Abstract

**Background:**

Geographic selection mosaics, in which species exert different evolutionary impacts on each other in different environments, may drive diversification in coevolving species. We studied the potential for geographic selection mosaics in plant-mycorrhizal interactions by testing whether the interaction between bishop pine (*Pinus muricata *D. Don) and one of its common ectomycorrhizal fungi (*Rhizopogon occidentalis *Zeller and Dodge) varies in outcome, when different combinations of plant and fungal genotypes are tested under a range of different abiotic and biotic conditions.

**Results:**

We used a 2 × 2 × 2 × 2 factorial experiment to test the main and interactive effects of plant lineage (two maternal seed families), fungal lineage (two spore collections), soil type (lab mix or field soil), and non-mycorrhizal microbes (with or without) on the performance of plants and fungi. Ecological outcomes, as assessed by plant and fungal performance, varied widely across experimental environments, including interactions between plant or fungal lineages and soil environmental factors.

**Conclusion:**

These results show the potential for selection mosaics in plant-mycorrhizal interactions, and indicate that these interactions are likely to coevolve in different ways in different environments, even when initially the genotypes of the interacting species are the same across all environments. Hence, selection mosaics may be equally as effective as genetic differences among populations in driving divergent coevolution among populations of interacting species.

## Background

The ecological outcomes of interactions between two species, such as mutualism and parasitism, often vary spatially among the different abiotic and biotic contexts in which those interactions occur; the result of this spatial variation in ecological dynamics is that the pattern of natural selection that species exert on each others' traits will vary among populations, that is, there will be a geographic 'selection mosaic' [1,2]. In addition, selection by species on each other may be strongly reciprocal in some populations, generating coevolutionary hotspots, and not in others, producing coevolutionary coldspots [3,4]. Finally, the processes of migration and gene flow among populations and genetic drift within populations may vary over space and time, influencing the distributions of species traits in each population [5-7]. Together, these processes result in a geographic mosaic of coevolution, which acts to generate and maintain much of the genetic and ecological diversity within and among populations of species [1,2].

Selection mosaics in species interactions result from geographic differences in how the fitness of one species depends on the distribution of genotypes in another species. Such geographic variation in selection can be driven by variation in both abiotic environmental factors, such as the nutrient content or physical composition of soils, and biotic factors, such as the species composition of the surrounding ecological community. Thus, a selection mosaic can be defined as a genotype-by-genotype-by-environment interaction (G × G × E) on fitness, in which variation in the 'environment' (E) can be abiotic or biotic [2,8,9]. Selection mosaics have now been suggested or characterized in a variety of different species interactions, including pines and birds [10], ants and wild cotton [11], camellias and weevils [12], and wild parsnips and parsnip webworms [13]. Most studies, however, have not been able to control for genotypes of the interacting species across environments to assess the strength of the G × G × E interaction.

Interactions between plants and mycorrhizal fungi have high potential to exhibit selection mosaics. Mycorrhizal fungi form a relationship with plants by colonizing the plant root system and extending their hyphae into the surrounding soil. Classically, this interaction has been considered a mutualism whereby fungal colonization greatly increases plant access to mineral nutrients in the soil, and the fungus receives organic nutrients synthesized by the plant [14,15]. In recent years, however, it has become evident that the ecological outcomes of plant-mycorrhizal fungus interactions are highly variable, ranging from mutualism to parasitism depending on a variety of biotic and abiotic environmental factors, especially ambient soil nutrient availability [16,17]. If environmental factors interact with plant and/or fungal genetics to change the outcome of plant-mycorrhizal interactions among populations, then selection mosaics could emerge as a consequence, driving the evolution of diversification in these interactions. Although the effects of individual biotic and abiotic factors on plant-mycorrhizal interactions have been fairly well characterized [15], it is currently not known whether these interactions, which are so pervasive in terrestrial ecosystems, exhibit evidence of selection mosaics. That question, however, is becoming important for our understanding of rapid evolution in terrestrial ecosystems as environmental conditions in many ecosystems are changing quickly and plants and their mycorrhizal fungi are being transported between continents [18].

In the work reported here, our goal was to explore the potential for selection mosaics in the interactions between bishop pine seedlings (*Pinus muricata *D. Don) and an ectomycorrhizal fungus (*Rhizopogon occidentalis *Zeller and Dodge) by experimentally varying lineages of the plant and fungus, as well as one biotic environmental factor (non-mycorrhizal soil microbes) and one abiotic environmental factor (soil composition), and measuring the variability in the performance of the plant and fungus. Non-mycorrhizal soil microbial communities may have a substantial impact on the colonization of roots by mycorrhizal fungi, and may alter the effects that mycorrhizal fungi have on plant growth [19]. For example, recent work has suggested that 'mycorrhizal helper bacteria' are present in soil, and that they are important for the success of the plant-fungus interaction [15,20,21]. Alternatively, rhizosphere bacteria may act to decrease the benefits conveyed by mycorrhizal fungi on plant growth [22]. Physical soil structure and composition may also have substantial impacts on the plant-mycorrhizal interaction. For example, Chen et al. [23] found much faster growth and higher ectomycorrhizal colonization of *Eucalyptus urophylla *seedlings grown in a laboratory potting soil mix compared with various field soils.

## Results

### Mycorrhizal colonization

**Figure 1:**
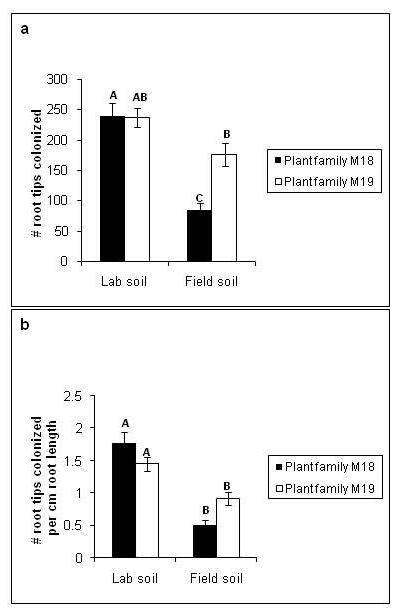
**Mycorrhizal colonization of *Pinus muricata *root tips**. Mycorrhizal colonization of *Pinus muricata *root tips colonized by *Rhizopogon occidentalis *as influenced by maternal seed family of *P. muricata *and soil type used. (a) Total colonization. (b) Root tips colonized per unit root length. Means with different letters are significantly different from each other (*p *< 0.05) according to Tukey HSD *post-hoc *tests.

On average, the mycorrhizal fungus *R. occidentalis *colonized 184.1 (± 10.1 standard error (SE), *n *= 128) root tips per plant and 1.16 (± 0.072 SE, *n *= 128) root tips per centimeter of root length. Total colonized root tips and root tips colonized per unit root length were both significantly affected by an interaction between plant maternal seed family and soil type (*F*_1,113 _= 7.66, *p *= 0.007). Specifically, total root tip colonization was approximately equal between the two seed families in the lab soil, but different between the two seed families in the field soil, with an overall trend towards lower colonization in the field soil (Figure 1a). When root tip colonization was standardized per unit root length, a similar result was observed, although the two plant families did not differ significantly from each other in either soil (Figure 1b, *F*_1,113 _= 10.53, *p *= 0.0015). Mycorrhizal colonization per unit root length also differed between the two fungal sporocarps (*F*_1,113 _= 3.95, *p *= 0.049; sporocarp 132: mean = 1.05 ± 0.093 SE, *n *= 64; sporocarp 133: mean = 1.28 ± 0.11 SE, *n *= 64). Neither the absolute levels of colonization by *R. occidentalis *nor the colonization by *R. occidentalis *per unit root length were related to the number of root tips colonized by contaminant fungi (covariate *p *= 0.374 and *p *= 0.841, respectively). Colonization by contaminant mycorrhizal fungi averaged 21.84 (± 1.97 SE, *n *= 128) root tips per plant and mostly did not differ among treatments, although contamination was significantly higher in the field soil than in the lab soil (*F*_1,113 _= 9.55, *p *= 0.0025; field soil: mean = 27.36 ± 2.78 SE, *n *= 64; lab soil: mean = 16.33 ± 2.63 SE, *n *= 64). Contamination on plants that were not inoculated with *R. occidentalis *averaged 44.75 (± 7.41 SE, *n *= 24).

### Root length

Plant root length averaged 175.1 cm (± 5.81 SE, *n *= 128) overall, and was influenced separately by both the type of soil used and the fungal sporocarp with which the plant was inoculated. Specifically, plants had a greater root length when planted in field soil than they did when planted in lab soil (*F*_1,112 _= 5.36, *p *= 0.022; field soil: mean = 193.9 ± 9.86 SE, *n *= 64; lab soil: mean = 156.3 ± 5.24 SE, *n *= 64). Plants inoculated with spores from fungal sporocarp 132 had a greater root length than those inoculated with spores from fungal sporocarp 133 (*F*_1,112 _= 4.29, *p *= 0.0406; sporocarp 132: mean = 186.9 ± 8.26 SE, *n *= 64; sporocarp 133: mean = 163.3 ± 7.95 SE, *n *= 64). Root length was also positively associated with colonization by contaminant mycorrhizal fungi (*F*_1,112 _= 16.64, *p *< 0.0001; regression slope = 34.12 ± 8.64 SE, regression intercept = -36.88 ± 10.59 SE).

### Response of root length to mycorrhizal inoculation

**Figure 2:**
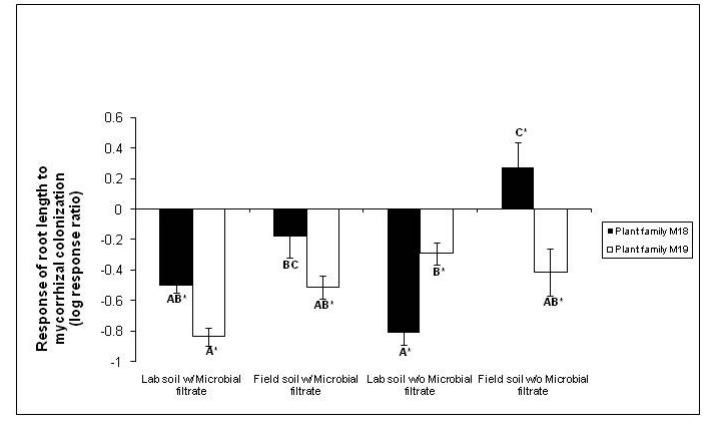
**Mean response of *Pinus muricata *root length to mycorrhizal colonization**. Mean response of *P. muricata *root length to mycorrhizal colonization (log response ratio = ln(*X*_*m*_/*X*_*n*_) where *X*_*m *_is the root length of inoculated plants and *X*_*n *_is the root length of non-inoculated plants), as influenced by a three-way interaction between *P. muricata *maternal seed family, soil type (lab versus field), and the addition of a microbial filtrate. Means with different letters are significantly different from each other (*p *< 0.05) according to Tukey HSD *post-hoc *tests, and means with an asterisk are significantly different from zero.

Overall, the response of root length to mycorrhizal inoculation was negative (mean LRR (log response ratio) = -0.409 ± 0.0474 SE, *n *= 128), and was influenced by a three-way interaction among plant family, soil type, and the presence/absence of microbial filtrate (Figure 2; *F*_1,112 _= 19.48, *p *< 0.0001). In both soil types, with microbial filtrate added, plant family M19 had a more negative response of root length to mycorrhizal inoculation than M18. Without microbial filtrate added, soil type affected the mycorrhizal response of the two plant families very differently: M19 exhibited a negative response regardless of the soil type, while M18 exhibited a negative response in lab soil and a positive response in field soil. The latter treatment combination (plant family M18 in field soil without microbial filtrate) was the only one to exhibit a positive response of root length to mycorrhizal inoculation (Figure 2). The response of root length to mycorrhizal inoculation also depended on which fungal sporocarp was used for inoculation (*F*_1,112 _= 4.12, *p *= 0.0447). Plants inoculated with spores from fungal sporocarp 133 had a more negative root length response to inoculation than those inoculated with spores from fungal sporocarp 132 (*F*_1,112 _= 4.29, *p *= 0.0406; sporocarp 132: mean = -0.332 ± 0.065 SE, *n *= 64; sporocarp 133: mean = -0.485 ± 0.0679 SE, *n *= 64). Finally, the response of root length to mycorrhizal inoculation was positively associated with colonization by contaminant mycorrhizal fungi (*F*_1,112 _= 20.17, *p *< 0.0001; regression slope = 0.248 ± 0.0572 SE, regression intercept = -0.268 ± 0.070 SE).

### Relative growth rate

**Figure 3:**
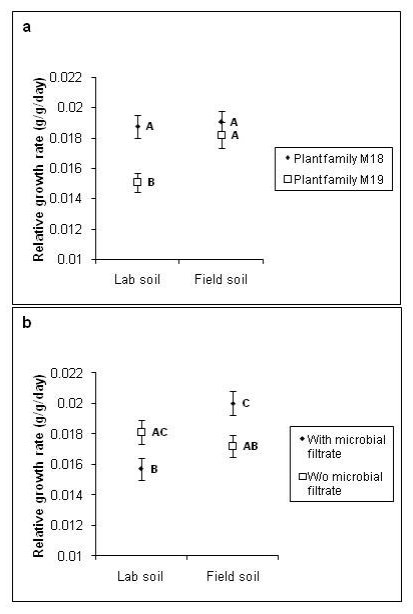
**Relative growth rate of *Pinus muricata***. (a) Mean *P. muricata *relative growth rate, as influenced by interaction between soil type and plant family. (b) Mean *P. muricata *relative growth rate, as influenced by an interaction between soil type and the presence or absence of microbial filtrate. Means with different letters are significantly different from each other (*p *< 0.05) according to Tukey HSD *post-hoc *tests.

Plant relative growth rate (RGR) averaged 0.0178 g/g/day (± 0.000391 SE, *n *= 128) and was significantly influenced by an interaction between plant family and soil type (F_1,113 _= 4.08, *p *= 0.0458). Specifically, plant family M19 had a lower RGR than family M18 in lab soil, but approximately the same RGR as M18 in field soil (Figure 3a). RGR was also significantly influenced by an interaction between microbial filtrate and soil type (*F*_1,113 _= 13.74, *p *= 0.0003). Specifically, in field soil the addition of the microbial filtrate increased RGR, while the opposite response to the microbial filtrate was observed in lab soil (Figure 3b). RGR was not associated with the number of root tips colonized by contaminant mycorrhizal fungi (*p *= 0.772).

### Response of relative growth rate to mycorrhizal inoculation

On average, RGR responded negatively to mycorrhizal inoculation (mean LRR = -0.0795 ± 0.0236 SE, *n *= 128), and the response was significantly more negative when plants were inoculated with microbial filtrate than when they were not (*F*_1,113 _= 4.53, *p *= 0.0356; with microbial filtrate: mean LRR = -0.1262 ± 0.03349 SE, *n *= 64, difference from zero: *p *< 0.0001; without microbial filtrate: mean LRR = -0.03284 ± 0.03257 SE, *n *= 64, difference from zero: *p *= 0.289). The two maternal seed families also differed in their response of RGR to mycorrhizal inoculation (*F*_1,113 _= 5.59, *p *= 0.0197; family M18: mean LRR = -0.1314 ± 0.03169 SE, *n *= 64, difference from zero: *p *< 0.0001; family M19: mean LRR = -0.02763 ± 0.03408 SE, *n *= 64, difference from zero: *p *= 0.372). Finally, the response of RGR to mycorrhizal inoculation was dependent on the type of soil used (*F*_1,113 _= 15.92, *p *= 0.0001). A significantly negative response to mycorrhizal inoculation occurred for plants growing in lab soil (mean LRR = -0.1671 ± 0.03146 SE, *n *= 64, difference from zero: *p *< 0.0001). In contrast, in field soil plants exhibited no significant response of RGR to mycorrhizal inoculation (mean LRR = 0.008044 ± 0.03190 SE, *n *= 64, difference from zero: *p *= 0.7946). Response of RGR to inoculation by *R. occidentalis *was not associated with the number of root tips colonized by contaminant mycorrhizal fungi (*p *= 0.393).

### Root:shoot ratio

**Figure 4:**
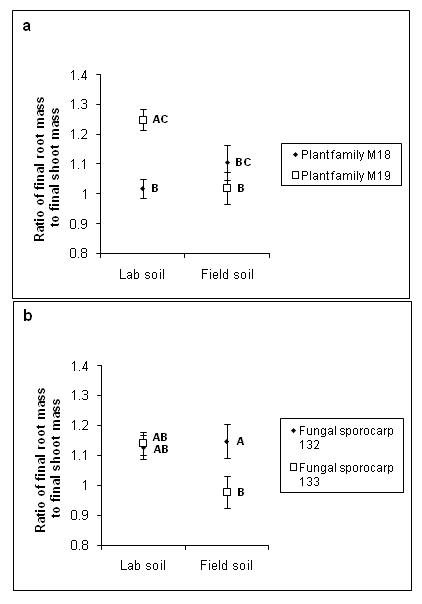
**Root:shoot ratio of *Pinus muricata***. (a) Mean *P. muricata *root:shoot ratio, as influenced by interaction between *P. muricata *maternal seed family used and soil type. (b) Mean *P. muricata *root:shoot ratio, as influenced by interaction between *R. occidentalis *sporocarp and soil type (lab versus field). Means with different letters are significantly different from each other (*p *< 0.05) according to Tukey HSD *post-hoc *tests.

Plant root:shoot ratio (overall mean = 1.098 ± 0.025 SE, *n *= 128) was affected by an interaction between plant maternal seed family and the soil type used (*F*_1,112 _= 13.58, *p *= 0.0004). In lab soil, plant family M19 had a greater root:shoot ratio than M18; in field soil, however, the root:shoot ratio of family M19 was not significantly different from that of family M18 (Figure 4a). The root:shoot ratio of the plants was also affected by an interaction between the fungal sporocarp used and the soil type (*F*_1,112 _= 4.19, *p *= 0.0430). Specifically, in lab soil the two sporocarps produced equal root:shoot ratios, whereas in field soil sporocarp 133 induced a significantly lower root:shoot ratio than sporocarps 132 (Figure 4b). Root:shoot ratio was positively associated with colonization by contaminant mycorrhizal fungi (*F*_1,112 _= 6.19, *p *= 0.0144; regression slope = 0.089 ± 0.037 SE, regression intercept = -0.096 ± 0.045 SE).

Additional file 1 contains summary statistics for all 8 response variables in the 16 different experimental treatment combinations. Additional file 2 contains the full statistical results (Wald *F*-tests from SAS PROC MIXED) of the separate univariate analyses of each of the eight response variables.

## Discussion

Our results demonstrate wide variability in a plant-mycorrhizal interaction in response to variation in both biotic and abiotic environmental factors, under conditions in which the same plant and fungal genotypes interact in every environment. Despite the inclusion of only four different combinations of plant and fungal lineages, the responses of plants and fungi to each other varied widely, and changed in response to different experimental soil environments. These results emphasize the contextual nature of ecological outcomes in such interactions, showing the potential for them to exhibit selection mosaics across landscapes, and highlighting the importance of exploring multiple conditions when evaluating the ecological outcomes and potential for evolution of interactions between plants and mycorrhizal fungi.

### Interactive effects of genotype and environment, and the potential for selection mosaics

The two experimental soil factors consistently interacted with plant or fungal lineages to influence plant and fungal performance in our experiments (Figures 1, 2, 3, 4). Fungal performance measures, in particular, demonstrate the potential for genotype-by-genotype-by-environment interactions (G × G × E) and thus selection mosaics. Root tip colonization by *R. occidentalis *(both total and per unit root length) was significantly influenced by an interaction between plant lineage and soil type (Figure 1), showing the potential for fungal fitness (assuming it is correlated with fungal colonization levels) to depend on both plant genotype and abiotic soil conditions. The experimental soil conditions we manipulated were not designed to match natural environmental variation among populations, and we specifically used plant and fungal lineages from only one population. Hence, our goal was not to demonstrate actual selection mosaics among natural populations of bishop pine and *R. occidentalis*. Rather, our goal was to evaluate the potential for selection mosaics, and to ask whether such mosaics, rather than initial genetic difference among populations, could potentially serve as the starting point for divergent selection on coevolving interactions.

### Effects of plant and fungal genetic variation, and potential coevolutionary selection

The existence of differences in symbiotic compatibility among the four combinations of plant and fungal lineages suggests the potential for ongoing coevolutionary dynamics between bishop pine and its mycorrhizal fungi at Pt. Reyes. For example, the two plant maternal seed families exhibited a more than four-fold difference in their response of RGR to mycorrhizal inoculation, with one exhibiting a significantly negative response. Not only does this result suggest significant genetic variation between plants for compatibility with mycorrhizal fungi, but it also may indicate that the relationship between the pine and fungus is not strictly a mutualism. Parasitic interactions are predicted to drive negative frequency-dependent selection between species, promoting genetic diversity [24]. Thus, our observation of parasitic effects of fungi on plants, as well as genetic variability among plants for response to fungi, shows the potential for negative frequency-dependent coevolutionary selection at a local scale. Alternatively, within-population genetic variability in a symbiosis could also be driven by gene flow from another population in which the pattern and intensity of coevolutionary selection differs from that at the study site. Neutral genetic diversity within and between populations of both *P. muricata *[25,26] and *R. occidentalis *[27] has been shown to be substantial, suggesting that processes such as genetic drift are not likely to limit the genetic variability available for coevolutionary selection.

The two fungal lineages differed in their impacts on plant growth, in a way that suggests that variation in compatibility between plants and fungi may sometimes be driven by feedbacks between plant and fungal growth responses. The colonization difference between the two fungal lineages provides an informative example of the interactive nature of plant and fungal genetic effects on symbiotic compatibility. Regardless of the plant family, fungal spore family 133 was found to have colonized more root tips per centimeter of root length than spore family 132. This result was driven by the fact that both fungal families actually colonized relatively similar absolute numbers of root tips, but plants growing with fungal spore family 132 produced greater root length (and a less negative response of root length to mycorrhizal inoculation) than plants colonized by fungal spore family 133. This result indicates that the variation between fungal lineages in intensity of mycorrhizal colonization may be due less to variation in fungal growth rates and more to variation in effects on plant growth, which feed back to influence mycorrhizal colonization intensity. Of course, this kind of feedback between plants and specific lineages of fungi would only be possible when individual plants are colonized by one or very few species or lineages of mycorrhizal fungi, as occurs in early succession after wildfires in *P. muricata *habitats [28]. The fact that the two fungal lineages colonized similar absolute numbers of root tips suggests that differences between the two sporocarps in average spore maturity or inoculum potential do not explain the variability observed.

### Implications for experimental tests and applications of plant-mycorrhizal interactions

Often, Pt. Reyes field soil elicited more variation in the outcome of the interactions between plant and fungus, compared with the lab soil (for example, Figures 1, 2, and 4b, but see also Figures 3 and 4a). For example, both total colonization by *R. occidentalis *and root:shoot ratio differed significantly between the two maternal seed families when grown in field soil, but did not differ when grown in lab soil. A potential reason for the observed increase in phenotypic responses in field soil may lie in the difference in texture of the two soil types. During the course of the experiment, we observed that the field soil did not retain water as well as the lab soil, most likely due to its lower organic matter content. Plants in field soil produced more root length than those in lab soil, perhaps to compensate for this lower water availability (Figure 2). Despite greater average root length in field soil, there were fewer total root tips colonized by *R. occidentalis *in the field soil compared with the lab soil (Figure 1a), a result consistent with recent experimental results for multiple ectomycorrhizal fungal species on bishop pine roots [29]. In contrast, contaminant mycorrhizal fungi, although rare overall, were more abundant in field soil than lab soil. As the contaminants we observed were always common greenhouse contaminants that disperse via airborne spores (likely a *Wilcoxina *sp. and a *Thelephora *sp.), and observed contaminants were never fungi such as *Rhizopogon *species that do not produce airborne spores, it is likely that contamination occurred via aerial spore deposition and all of our experimental treatments received approximately equal input of spores of contaminant fungi. Thus, the higher level of contaminant colonization in field soil compared with potting mix likely reflects a response by the contaminant fungi to the differing conditions in those treatments.

Furthermore, the difference between soil types in colonization by *R. occidentalis *was much more pronounced for one plant lineage than the other (Figure 1a). This combination of results suggests that the reduced water availability in field soil may have resulted in a more stressful environment in which genetic variability in the plant-fungus interaction was more likely to be expressed. In general, stronger differences were observed *between *plant and fungal families in their responses to the field soil compared with the lab soil, despite the probability that there was more heterogeneity in soil conditions among field soil pots *within *each plant-fungus treatment combination compared with lab soil. The field soil was screened to remove large debris and was mixed to homogenize it, but still contained significant heterogeneity compared with the lab soil.

Fungal performance did not appear to directly depend on the presence or absence of a non-mycorrhizal microbial community added as a filtrate. At first glance, this result appears to be in contrast to several studies suggesting that the soil microbial community is an important third member in the mycorrhizal-plant relationship [21]. Our results, however, may simply represent one of a range of results that can occur in these interactions. We used a single species of fungus, and the soil microbial filtrate was composed of organisms of unknown identity and number. Bowen and Theodorou [30] found fungal species-specific reactions to different bacteria isolated from soil. The specificity they found was between species, whereas our study focused on within-species variation. The apparent lack of response of *R. occidentalis *in our study to the non-mycorrhizal microbial community could be due to the presence of a wide array of genotypes in that community, having a diversity of effects on the different fungal genotypes in our experiment. Regardless, laboratory measurements of colonization by *R. occidentalis *may not be very dependent on the presence of non-mycorrhizal microbes.

In contrast, plant performance and the response to mycorrhizal inoculation were influenced by augmentation of the non-mycorrhizal microbial community. With the addition of the microbial filtrate, root length was consistently greater in non-mycorrhizal controls than in mycorrhizal treatments, especially for plant family M19 (Figure 2). This observation may indicate that when the microbial filtrate was added, plants generally experienced reduced access to soil nutrients in the absence of mycorrhizal fungi and responded by increased root allocation. In contrast, without the microbial filtrate, one plant family × soil combination exhibited a positive response of root length to mycorrhizal inoculation (Figure 2).

The response of plant RGR to mycorrhizal inoculation was significantly negative in the presence of the microbial filtrate, and was neutral in its absence. The reason for this result is not readily apparent, and is in contrast to findings by Heinonsalo et al. [19], which showed that shoot volume of mycorrhizal inoculated Douglas fir seedlings was higher than in control treatments regardless of bacterial inoculation. Furthermore, we found that addition of the microbial filtrate had opposite effects on plant RGR depending on whether plants were growing in lab versus field soil (Fig. 3b), suggesting that the impact of non-mycorrhizal microbes on plants may be strongly contingent on the abiotic environmental context.

Numerous previous studies have also demonstrated significant genetic variability within plant and/or fungal species for symbiotic compatibility in mycorrhizal interactions. For example, in an inoculation study utilizing 20 *Pisolithus *isolates, Burgess et al. [31] found that *Eucalyptus grandis *varied greatly in its growth response to the different fungal genotypes. Similarly, studies of within-population compatibility in plant-*Rhizobium *interactions have also found significant variability in performance. Estaún et al. [32] found substantial differences among pea genotypes in their responses to different species of arbuscular mycorrhizal fungi, ranging from positive to neutral. As discussed by Trappe [33] three decades ago, such genetically based variation in compatibility between the partners in a putative mutualism points to the need to consider plant and fungal genotypes as factors in inoculations for forestry and nursery production. When using a limited number of plant or fungal genotypes, practitioners may be reducing the chances of seedling success. Our results highlight the need not only to consider plant and fungal genetic variation, but also their interactions with biotic and abiotic environmental factors.

The variation found in these experiments is likely to be modified within natural populations, where each interaction between a plant species and a fungal species is often part of a larger network of interactions, and in which plant roots and mycorrhizal fungi are not restricted within the artificial conditions of a pot. Ectomycorrhizal fungus communities are typically diverse, with multiple species colonizing the roots of individual trees simultaneously [34], and two or more plant root systems can be interconnected by a common mycorrhizal network [35] with the potential to transfer nutrients among the plants (see, for example, [36]). In addition to the variation we observed in compatibility among different genetic combinations of plants and fungi, there may also be variation among plants or fungi in their response to mycorrhizal networks or ectomycorrhizal fungus community composition. For example, a particular plant-fungus combination may exhibit low performance when compared with other combinations in the laboratory, but may exhibit superior performance in the context of a diverse community and the potential to connect with mycorrhizal networks.

## Conclusion

We found significant genetic variation for symbiotic compatibility within the Pt. Reyes population of bishop pine and the ectomycorrhizal fungus *Rhizopogon occidentalis*, as well as substantial dependence of the plant-fungal interaction on variation in biotic and abiotic experimental soil characteristics. This variation in plant and fungal responses to experimental conditions illustrates the broad plasticity of the interaction, and the potential for mycorrhizal interactions to exhibit geographic selection mosaics across landscapes, as abiotic and biotic factors vary and induce corresponding changes in the impacts that species have on each other.

## Methods

We tested for the overall and interactive effects of plant lineage, fungal lineage, and two environmental factors (soil type and the presence or absence of a non-mycorrhizal soil microbial assemblage) on pine seedling performance and mycorrhizal colonization of seedling roots by growing bishop pines from seeds in individual pots in a growth chamber. We employed a completely randomized 2 × 2 × 2 × 2 factorial experimental design, using maternal half-sib families of seeds from two different individual bishop pine trees, spores from two different *R. occidentalis *fungal sporocarps (full-sib families of spores), two soil types (a commercial lab potting mix to which we refer hereafter as 'lab soil' or field-collected 'field soil'), and the addition of a microbial filtrate from non-sterilized field soil in some treatments. Each of the 16 treatment combinations was replicated eight times (n = *8*), for a total of 128 pots. In addition, for the purpose of calculating plant response to mycorrhizal inoculation, each of the eight treatment combinations of maternal seed family, soil type, and microbial filtrate had three replicates (*n *= 3) of a corresponding 'no fungus' treatment that was not inoculated with mycorrhizal fungi, for an additional 24 pots.

### Preparation of pine seedlings and mycorrhizal fungus inoculum

Mycorrhizal fungus spores from two different fungal sporocarps of *R. occidentalis *were collected from beneath bishop pines (greater than 50 m apart to insure collection from separate fungal genets) in the Mount Vision area of Pt. Reyes National Seashore (Marin County, California, USA, N38 03.46' W122 14.92') in December 2004. Fungal sporocarps were coarsely chopped and refrigerated (at 4°C) in tap water for 1 month, and then a spore slurry was prepared by blending the sporocarp material with de-ionized water. The two slurries were then diluted to ~6.25 × 10^7 ^spores/ml and stored at 4°C. Portions of each sporocarp were saved and dried separately, and have been deposited in the Pullen Herbarium at the University of Mississippi.

Seeds from two different maternal families of bishop pine were extracted from cones collected in the Mt. Vision area of Pt. Reyes National Seashore in December 2003. For use in the experiment, they were surface-sterilized by soaking in a 1% bleach solution for 5 minutes, followed by extensive rinsing in both tap water and de-ionized water. The seeds were then soaked in water at 4°C for 48 hours, patted dry, and stored in moist paper towels at 4°C for 3 weeks. After stratification, the seeds were sown in a sterile peat-vermiculite mixture and placed in a growth chamber for germination. We deliberately chose two maternal families of seeds with similar average seed mass (family M18 mean = 0.0119 g, SD = 0.0022, *n *= 10; family M19 mean = 0.0157 g, SD = 0.0018, *n *= 10) to minimize potential effects of maternal environment on seedling growth rates and other performance measures.

### Preparation of microbial filtrate and experimental soil media

Field soil was collected in June 2005 by removing the upper 15 cm from multiple patches of soil within the same bishop pine stand where the sporocarps and seeds were collected. The field-collected soil was divided into two 5-gallon buckets and returned to the laboratory, where each was filled with tap water. The soil was allowed to soak for 2 hours, after which the liquid was drained, and passed via vacuum filtration through a 5 μm nylon mesh screen to remove mycorrhizal fungus spores. The filtrate from the two buckets was combined and stored at 4°C to be used later for the microbial filtrate treatment. The field soil was then prepared for use in the experiment by sifting over a 2 mm sieve to remove large debris (such as branches, pine cones, and rocks) and then mixed thoroughly to reduce heterogeneity among pots. Both the field soil and the lab soil (Promix PGX, a peat/vermiculite/limestone mixture with added macro- and micro-nutrients; Premier Horticulture, Inc., 1785 55th Avenue, Dorval, Quebec, Canada H9P 2W3) were then autoclaved at 121°C for 3 hours. The soil at the field collection site in the Mt. Vision area of Pt. Reyes National Seashore is classified as part of the Inverness Loam series, which is a fine-loamy, mixed, active, isomesic Ultic Haplustalf. In the field, it has a moderately low pH (5.1–6.0), a bulk density of 0.66–1.5 g/cc, and 2–4% organic matter (USDA Soil Survey, Marin County, CA; [37]). The lab potting soil, Promix PGX, has a similar pH (5.0–6.5), lower bulk density (0.13–0.16 g/cc), and a much higher organic matter content (50–60%) compared with the field soil (Premier Horticulture, Inc.). Previously, we found that Promix potting soils did not exhibit substantial changes in nutrient availability in response to autoclaving, with extractable P and K actually decreasing slightly in response to autoclaving and no evidence of N or C volatilization (unpublished data). Many forest soils, however, are known to exhibit increases in nutrient availability in response to autoclaving treatments, with these increases being similar to those caused by heating treatments designed to mimic those caused by wildfires [38,39]. As the ecological context in which *P. muricata *seedlings and *Rhizopogon *species interact most directly is in post-wildfire soils, our autoclaved experimental soils are not likely to be much less realistic as a growth medium compared with non-autoclaved experimental soils.

### Experimental set-up

After 3 months the two different maternal families of seedlings were transplanted to pots (5.0 cm diameter × 17.5 cm deep) and the 16 different experimental treatment combinations were initiated. Half of the pots were filled with autoclaved field soil, and the other half with autoclaved lab soil. The microbial filtrate treatment was applied to half of these pots by pipetting 10 ml of the filtrate onto the surface. Mycorrhizal inoculations were performed by pipetting 1 ml of mycorrhizal spore slurry (containing ~6.25 × 10^7 ^spores) onto the surface. Each pot was topped with a layer of sterile sand to avoid splashing of spores or bacteria during watering, which took place twice a week using de-ionized water. The plants were kept in a fluorescently lit growth chamber at 60% humidity with 14-hour days (with around 225 μmol m^-2 ^s^-1 ^of light at plant height) at 23°C and 10-hour nights at 10°C. The pots were distributed in racks randomly with respect to treatment, with re-randomization every 6 weeks throughout their growth period.

### Data collection

After 22 weeks, the seedlings were removed from the pots, and the soil gently rinsed from the roots. Roots were separated from shoots, and total root length was estimated using the grid-line intercept method [40]. All root tips were examined, and the number of pine root tips colonized by *R. occidentalis *was counted, as well as the number of root tips colonized by contaminant morphotypes of mycorrhizal fungi. Two different contaminant morphotypes were observed and were generally rare, occupying approximately 10% of all colonized root tips. Colonization by *R. occidentalis *was analyzed on an absolute basis per plant, as well as relative to total root length to control for effects of final plant size and available root colonization sites.

Both the roots and the shoots were placed in a drying oven at 60°C for 48 hours, after which the dried roots and shoots were weighed separately. Final root length, root:shoot ratio, and total estimated RGR were used as measures of plant performance. At the time of initial mycorrhizal inoculation, we measured the length of the needle-bearing stem on each plant, a measurement we have found previously to be predictive of total dry mass. As in Hoeksema and Thompson [41], we then used a previously established regression equation of total dry mass (in grams) on needle-bearing stem length (green length, in millimeters) to estimate total dry mass of each plant at the time of inoculation (ln(mass) = ln(green length) × 1.97 – 7.823). RGR from inoculation to the end of the experiment was estimated as (ln(*m*_2_)-ln(*m*_1_))/(number of days), where *m*_1 _is estimated total dry mass at time of inoculation and *m*_2 _is measured total dry mass at the end of the experiment. For root length and RGR, we also calculated the relative response to mycorrhizal inoculation, by comparing performance (RGR or root length) in inoculated replicates to the mean performance of the three non-inoculated replicates for each treatment group. Specifically, for each inoculated replicate, we calculated a log response ratio (LRR) of RGR and root length:

LRR = ln(*X*_*m*_/*X*_*n*_)

where *X*_*m *_is the performance (RGR or root length) in mycorrhizal inoculated pairings and *X*_*n *_is the mean performance of the three non-inoculated replicates. This metric is positive when a positive response to mycorrhizal inoculation is observed, and negative when a negative response to mycorrhizal inoculation is observed. We chose to use the LRR because it provides a relative measure of response to mycorrhizal inoculation, and is linear with respect to variation in the numerator and the denominator. Also, LRRs have been determined to have particularly favorable properties for any subsequent meta-analyses, compared with other comparable metrics such as the standardized mean difference [42].

Collectively, our measures of pine seedling performance provide complementary information on plant growth and the outcome of interactions with other species. RGR is thought to be an important measure of plant performance, as it integrates a variety of plant physiological components and is independent of plant size [43,44]. Root length may be an indicator of competitive ability for soil resources [45]. We also calculated plant root:shoot biomass ratio because it is often predicted to vary significantly among plants depending on the relative importance of limitation by aboveground and belowground resources [46-48]. In general, early performance of seedlings has been found to be an important predictor of later success in field studies of demography in pine populations [49] and a variety of other species (see, for example, [50] and the review in [51]). In addition, evidence from at least one forest system suggests that the functional traits of trees are more adapted to their early successional environments (that is, the regeneration niche) than to their late successional environments [52]. In early succession following wildfires, individual *P. muricata *seedlings are usually colonized by only one or two ectomycorrhizal fungal species, mostly members of the genus *Rhizopogon *and a few species of Ascomycetes including *Wilcoxina *species and *Tomentella sublilacina *[28]. Thus, measurements of *P. muricata *seedling growth and response to *R. occidentalis *in different environments should be somewhat correlated with *P. muricata *fitness and the probability of reaching the age of reproduction.

It is inherently problematic to quantify the fitness of clonal soil microbes such as mycorrhizal fungi (see [53] for a discussion of the problem). Nevertheless, ectomycorrhizal fungi such as *Rhizopogon *are obligate symbionts on their host plants, and thus the total extent to which they colonize their hosts is expected to correlate with their ability to obtain fixed carbon for growth and sexual reproduction. When controlling for the size of the host plant's root system, the extent of fungal colonization is expected to reflect overall compatibility between the host plant and the fungus. Thus, although we could not directly measure fitness of the plants and fungi in these experiments, our measures of success are likely to be closely correlated with fitness, and thus informative for arguments about coevolution and adaptation [53].

### Data analysis

We analyzed eight response variables in separate analyses: total root tips colonized (by the target fungus *R. occidentalis*), root tips colonized per centimeter of root length, total contaminant-colonized root tips, root length, LRR of root length to mycorrhizal inoculation, RGR, LRR of RGR to mycorrhizal inoculation, and root:shoot ratio. We analyzed our data with four-way ANOVA using the MIXED procedure in SAS v. 9.1 (SAS Institute Inc., Cary, NC, USA). The four independent variables were soil type (lab soil or field soil), maternal seed family (M18 or M19), fungal sporocarp (132 or 133) and microbial filtrate (presence or absence). For all of the response variables besides contaminant-colonized root tips, the number of contaminant-colonized root tips was initially included as a covariate. This covariate was eliminated from statistical models when it was found to be highly non-significant (*p *> 0.37). When the covariate was significant, its relationship with the response variable was explored using simple linear regression of residuals (from the model lacking the covariate) on the covariate. The normality of all response variables was assessed by inspection of histograms of residuals, and one variable (contaminant-colonized root tips) was log-transformed to achieve normality. All four-way interactions were found to be highly non-significant (*p *> 0.25), and were excluded from all final statistical models; this result was consistent with our expectations, since we designed the study only to have power to examine three-way interactions. For all significant (*p *< 0.05) statistical interactions we tested for differences among individual treatment groups using *post-hoc *Tukey HSD (honestly significantly different) comparison of means. For the two variables that measured response to mycorrhizal inoculation, we also tested whether individual means for treatment combinations were significantly different from zero, using *t*-tests.

## Authors' contributions

BJP participated in planning the study, took primary responsibility for executing the experiment and collecting the data, and took the lead in interpretation of the data and drafting of the manuscript. JDH participated in planning the study, participated in executing the experiment and collecting the data, performed the data analysis, and contributed to interpretation of data and writing of the manuscript. JNT participated in planning the study, interpretation of data, and writing the manuscript. All authors read and approved the final manuscript.

## Supplementary Material

Additional file 1 **Summary statistics**. Additional file 1 is an Excel file (Summary_stats.xls) containing summary statistics for all 8 response variables in the 16 different experimental treatment combinations. Click here for file

Additional file 2 **Statistical analysis results**. Additional file 2 is an Excel file (Statistics.xls) containing the full statistical results (Wald *F*-tests from SAS PROC MIXED) of the separate univariate analyses of each of the eight response variables. Click here for file
